# Factors associated with changes in the quality of life and family functioning scores of primary caregivers of children and young people with primary brain tumors in Karachi, Pakistan: a prospective cohort study

**DOI:** 10.1186/s12887-024-04867-z

**Published:** 2024-06-08

**Authors:** Nida Zahid, Syed Ather Enam, Thomas Mårtensson, Iqbal Azam, Naureen Mushtaq, Mariya Moochhala, Faiza Kausar, Aneesa Hassan, Saqib Kamran Bakhshi, Farrukh Javed, Lal Rehman, Muhammad Nouman Mughal, Sadaf Altaf, Salman Kirmani, Nick Brown

**Affiliations:** 1 Department of Surgery, Aga Khan University, Karachi, Pakistan; 2 Global Health and Migration Unit Department of Women’s and Children’s Health, Uppsala University, Box 256, Uppsala, 751 05 Sweden; 3 Department of Community Health Sciences, Aga Khan University, Karachi, Pakistan; 4 Department of Pediatric Oncology, Aga Khan University, Karachi, Pakistan; 5 Department of Psychiatry, Aga Khan University, Karachi, Pakistan; 6 Department of Neurosurgery, Jinnah Post graduate Medical Centre, Karachi, Pakistan; 7 Division of Women & Child Health, Aga Khan University, Karachi, Pakistan; 8 Department of Pediatrics, Aga Khan University, Karachi, Pakistan

**Keywords:** Quality of life, Family functioning, Primary caregivers, Mothers, Children and young people, Primary brain tumor, Cohort study, Pakistan

## Abstract

**Background:**

There are limited data available, particularly in low- and middle-income countries (LMICs), on the long-term quality of life (QoL) and family functioning of primary caregivers of children and young people (CYPs) affected by primary brain tumors (PBTs). This study aimed to assess the factors associated with the mean change in QoL and family functioning scores of primary caregivers of CYP patients with PBTs 12 months posttreatment.

**Methods:**

This prospective cohort study enrolled CYPs aged 5–21 years with newly diagnosed PBTs and their primary caregivers. The study was carried out between November 2020 and July 2023. The primary caregivers of CYPs were recruited from two major tertiary care centers in Karachi, Pakistan. The primary caregivers QoL were assessed by the Pediatric Quality of Life Inventory (PedsQL) Family Impact Module. The assessment was undertaken by a psychologist at the time of diagnosis and 12 months posttreatment. The data were analyzed with STATA version 12.

**Results:**

Forty-eight CYPs with newly diagnosed PBTs and their primary caregivers (46 mothers and 2 fathers) were enrolled. At 12 months posttreatment, 25 (52%) CYPs and their primary caregivers (mothers) were reassessed, and 23 (48%) were lost to follow-up. On multivariable analysis, a significant decrease in mothers’ mean 12-month posttreatment QoL and family functioning scores was associated with CYP having posttreatment seizures (beta= -10.2; 95% CI: -18.4 to -2.0) and with the financial burden associated with the CYP’s illness (beta= -0.3; 95% CI: -0.4 to -0.1). However, in those cases where CYP had higher posttreatment quality of life scores (beta = 0.4; 95% CI = 0.1, 0.6) and posttreatment higher verbal intelligence scores (beta = 0.1; 95% CI = 0.01, 0.3), the mothers’ QoL and family functioning scores were significantly greater.

**Conclusion:**

We found a significant decrease in QoL of mothers who had a high financial burden and whose CYP had posttreatment seizures. However, those whose CYPs had higher posttreatment verbal intelligence scores and quality of life scores had significantly greater QoL scores. Identification of the factors that influence primary caregivers QoL has the potential to aid in the development of targeted strategies to alleviate stressors and improve the overall quality of life for primary caregivers and their children who are at high risk.

**Supplementary Information:**

The online version contains supplementary material available at 10.1186/s12887-024-04867-z.

## Background

Cancer in children and young people (CYPs) is a stressful event that affects the entire family and has long-lasting psychosocial effects on survivors and their caregivers. Due to improvements in treatment, a greater fraction of CYPs, including those with primary brain tumors (PBTs), survive cancer of all types [1]. The behavioral science literature shows that stress and coping prove challenging for all parents to some extent [2]. Parents of cancer patients endure continuous uncertainty and long-term concerns about the future health of CYP, and the risk of cancer recurrence and care for these patients can be very demanding [3, 4]. However, caring for PBT survivors involves numerous challenges that may differ from caring for survivors of other cancer types. These challenges are due to the need for additional neurotoxic treatments, differing prognoses, and increased risk of late effects [5]. 

The long-term consequences of PBT have been reported to predict parental quality of life (QoL) and family functioning. Klassen AF et al. reported that these patients experience higher stress levels and a diminished QoL [6]. Other studies have shown that parental QoL can be influenced by their CYP disease status, psychological factors [7–12] and parental characteristics [6, 7, 12–14]. The high levels of distress experienced by parents underscore the importance of early intervention, such as cognitive behavioral therapy and psychoeducation, for high-risk individuals [11, 15]. Screening of primary caregivers and referral of those at high risk to mental health services may improve their psychological outcomes [16]. Targeted support and coping therapies have been proven to increase well-being in parents in the hospital setting [17]. 

Although there are some studies on QoL in CYP patients with PBTs, there are very few longitudinal data available for their caregivers [7, 18, 19]. There is currently limited research from low- and middle-income countries (LMICs) on the assessment of parental caregiver QoL and family functioning. A single qualitative study in Pakistan assessing the factors affecting the well-being of adult brain tumors suggested that the role of the family, particularly in the Pakistani context, is pivotal [20]. 

Our prospective cohort study aimed to determine the factors associated with the mean change in QoL and family functioning scores for primary caregivers of CYP patients diagnosed with PBTs 12 months posttreatment at two tertiary care hospitals in Karachi, Pakistan.

## Methods

### Study design and setting

A prospective cohort study was chosen because it allows the examination of changes over time within the same group of individuals. The study was conducted in Karachi, Pakistan, at two tertiary care centers: the private tertiary care hospital Aga Khan University Hospital (AKUH) and the public tertiary care hospital Jinnah Postgraduate Medical Center (JPMC). The data were collected over 33 months from November 2020 to July 2023.

### Study population and eligibility criteria

The CYPs aged 5 to 21 years with a diagnosis of PBTs and their primary caregivers of all the patients living in Pakistan, presenting at any tumor stage, without previous treatment and pre-existing debilitating diseases, were included. Those who were unable to communicate in English or Urdu were excluded. The details of the eligibility criteria of the CYP with PBTs are given in the study conducted by our research group [21].

### Sample size

The sample size estimation was based on parallel studies conducted by our research group with the primary objectives of evaluating 12-month posttreatment changes in neurocognition [21] and QoL scores (the results of the study are under review) in CYPs with PBTs. A standard estimation formula was used, where *n* = 8(CV^2^)/(PC^2^) [1+ (1-PC) ^2^], with PC representing the proportionate change in means (PC = (µ0 − µ1)/µ0) and CV indicating the coefficient of variation (CV = σ0/µ0 = σ1/µ1) [22]. A minimum of 48 CYPs with PBTs were required to achieve 80% statistical power and to detect a minimum 10% change in mean neurocognition and QoL scores, as well as a 20% or less change in the coefficient of variation [23–25] at a two-sided 5% level of significance. The sample size was inflated by 65% to account for potential loss to follow-up or nonresponse, figure based on prior research that aimed to assess the epidemiology, treatment, and outcomes of children with brain tumors in a single tertiary care center in Pakistan. Nearly half of the patients were lost to follow-up [26].

### Data collection

A nonprobability purposive sampling method was employed to select the study participants. A research assistant screened the potential CYPs with PBTs during scheduled appointments at surgical/oncology clinics at AKUH and JPMC. Forty-eight CYP with PBTs were eligible and were recruited along with their primary caregivers (46 mothers and 2 fathers). The same research assistant (RA) was responsible for collecting data at both study sites. For screening and recruitment of the participants, the research assistant followed the processes rigorously outlined in the standard operating procedures (SOPs). Training sessions for the RA were conducted by the principal investigator (PI) at both the centers to ensure proficiency in executing procedures accurately. The PI had regular meetings with the RA to discuss issues and ensure alignment on procedures. The RA maintained detailed documentation of activities at each center. The PI conducted periodic onsite visits to observe the processes and provide feedback to the RA. The assessment of CYP and their primary caregiver took place at two points: pretreatment, i.e., at the time of PBT diagnosis, and subsequently, at 12 months posttreatment. For those CYP whose first intervention was surgery, reassessment was performed post-surgery, while for those whose primary treatment was adjuvant therapy, reassessment took place after the completion of adjuvant therapy. However, for CYP patients who underwent no intervention, the assessment was conducted 12 months after the time of diagnosis.

### Independent variables

#### CYP Sociodemographic, tumor and treatment factors

The following data were collected from the CYPs: (1) demographic data and (2) tumor and treatment information, which included tumor histopathology, tumor location (supratentorial, infratentorial, suprasellar and sellar), history of pretreatment seizures, presence or absence of hydrocephalus at diagnosis confirmed radiologically by magnetic resonance imaging (MRI), posttreatment seizures, and type of treatment (surgical resection, chemotherapy, radiotherapy, and shunt placement). Surgical tumor resection types were determined by institutionally established cut-offs, assessed post-MRI, and categorized as total resection (100% removal), maximum safe resection (> 90% removal prioritizing safety), or subtotal resection (< 90% removal). This information was extracted by the RA from the surgeons’ notes in the patients’ files and further confirmed by the surgeon.

#### Parental sociodemographic factors and financial burden

The data were also collected on the parents’ sociodemographic and financial burden. The financial burden was assessed by a visual analog scale (VAS), which ranged from 0 (representing no financial burden) to 100 (representing high financial burden). There is substantial literature demonstrating the reliability of visual analog scale (VAS) assessments, specifically in terms of interrater reliability and test-retest reliability, as described by Brazier et al. [27] Parents were also asked about receiving financial aid for CYP treatment, which could come in the form of assistance from AKUH for poor patients, zakat (a form of almsgiving or charity in Islam), insurance coverage, or support from their extended families. Additionally, parents were surveyed about any unforeseen hospitalizations for CYP, admissions to the intensive care unit, or any instances of parents losing their jobs or having to quit their job [28]. 

#### Quality of life of CYP patients

The QoL for CYP patients was evaluated with the PedsQL 4.0, a validated generic core scale and brain tumor module that is applicable both internationally and in the local language of Pakistan, Urdu [29, 30]. A parallel study by our research group examining the QoL of CYP is under review.

#### Neurocognition of CYP

The verbal and nonverbal neurocognition outcomes of CYP patients were assessed using validated tools. Verbal intelligence was determined with the Slosson Intelligence tool, Revised 3rd edition (SIT-R3) [31]. Perceptual reasoning was assessed by Raven’s Progressive Matrices (RPM) tool [32, 33]. Processing speed was assessed using the Wechsler Intelligence Scale for Children (WISC-V) [34–36] and Wechsler Adult Intelligence Scale (WAIS-IV) [37]. For consistency reasons only one research assistant (assessor), a trained junior psychologist who was well versed with the tools, was responsible for conducting neurocognitive assessments. She conducted the assessments in a separate room, each lasting for 1 to 1.5 h. The details of the study conducted by our research group, assessed the predictors of neurocognition outcomes in children and young people with primary brain tumors are given in the following publication [21].

### Outcome variable

#### Parental health-related quality of life and family functioning

Health-related QoL and family functioning of the primary caregivers were evaluated using an internationally validated tool, the Pediatric Quality of Life Inventory (PedsQL)™ Family Impact Module. This tool consists of 36 items distributed across 6 scales measuring parental self-reported functioning, including physical functioning (6 items), emotional functioning (5 items), social functioning (4 items), cognitive functioning (5 items), communication (3 items), and worry (5 items). Additionally, there are 2 scales measuring parent-reported family functioning: Daily Activities (3 items) and Family Relationships (5 items) [38]. It is a 5-point response scale with (0 = never a problem; 4 = always a problem). Items are reverse-scored and linearly transformed to a 0–100 scale (0 = 100, 1 = 75, 2 = 50, 3 = 25, 4 = 0), so that higher scores indicate better functioning (less negative impact). Scale Scores are computed as the sum of the items divided by the number of items answered (this accounts for missing data). If more than 50% of the items in the scale are missing, the Scale Score is not computed [39]. 

The PedsQL Family Impact Module Total Scale Score is the sum of all 36 items divided by the number of items answered. The Parent HRQOL Summary Score (20 items) is computed as the sum of the items divided by the number of items answered in the Physical, Emotional, Social, and Cognitive Functioning Scales. The Family Functioning Summary Score (8 items) is computed as the sum of the items divided by the number of items answered in the Daily Activities and Family Relationships Scales [39]. 

As the tool has not previously been validated in Urdu, content validation was conducted with the input of a panel of experts, including neurosurgeons, psychologists, mental health nurses, pediatricians and neurologists. Each expert rated the tool for relevance and clarity. Based on these ratings, the content validation index (CVI) was determined, resulting in a CVI of 0.7 for clarity and 0.9 for relevance indicating acceptable to excellent agreement on both clarity and relevance, respectively. The internal consistency (reliability) of the total score, which included 36 items, as well as parental health-related QoL (28 items) and family summary score (8 items), was excellent, with Cronbach’s alpha values ranging from 0.91 to 0.93. The internal consistency of the individual modules ranged from acceptable to excellent, with Cronbach’s alpha values varying from 0.52 to 0.94. The greatest floor effects were observed for emotional functioning, while the greatest ceiling effect was observed for family relationships (Supplemental Table 1).

### Ethical considerations

The study received ethical and institutional review committee approval from the relevant sites AKUH-ERC (ERC#2020-4859-11855) and JPMC (F2-81/2021-GENL/65,706/JPMC). Informed consent was obtained from the parents. For CYPs aged 18 years and older, informed consent was directly obtained. For those under 18 years of age, informed assent and parental consent were obtained in both English and Urdu based on the participants’ understanding. The interviews were conducted in a private room to ensure confidentiality. Since this was an observational study, no intervention was given to the participants. However, those participants with a significant decline in QoL were guided by the psychologist towards a treatment plan.

### Statistical analysis

The analysis was performed with STATA version 12 software (STATA Corp., Texas). Quantitative variables are presented as mean and standard deviation (SD) or median with interquartile range (IQR) for normal and nonnormal data, respectively. The mean differences between mothers’ QoL and family functioning scores before treatment and 12 months after treatment were assessed using paired t tests. Categorical variables are expressed as frequencies and percentages. To explore the correlation between the QoL scores of primary caregivers and their CYPs, Pearson and Spearman correlation coefficients were estimated. To determine the relationships of various independent factors, including tumor factors, treatment factors, psychological factors, and parental characteristics, with mothers’ QoL and family functioning scores, generalized estimating equations were employed while adjusting for covariates. Unadjusted and adjusted beta coefficients with 95% confidence intervals are reported. Additionally, potential interactions were examined. *P* values less than 0.2 for univariate analysis and *p* values less than 0.05 for multivariable analysis were considered statistically significant. We assessed the confounding factors such as socioeconomic status (SES), tumor grade and age in regression analysis. The cutoff criteria for determining the significance of confounding variables were > 15% to see the change in beta coefficient.

## Results

Forty-eight CYP with PBTs were eligible and were recruited along with their primary caregivers (46 mothers and 2 fathers). However, at 12 months post-treatment 23 CYP and their primary caregivers were loss to follow-up and we were able to assess only 25 CYP and their primary caregivers, who were the mothers.

### CYP demographic, tumor and treatment factors

A total of 48 CYPs with PBTs were enrolled. The mean age was 12.8 ± 4.6 years, and 60% of the patients were males. Seventeen (35%) patients had an infratentorial tumor. Pilocytic astrocytoma was the predominant type of astrocytoma in 12 (25%) patients. Hydrocephalous and shunt placement was performed in 23 (48%) of the CYPs. Posttreatment seizures occurred in 4/25 (16%) patients. A total of 26 (54%) patients underwent surgical resection only, 12 (25%) underwent surgical resection with adjuvant therapy (chemotherapy and/or radiotherapy), 1 (2%) had only radiotherapy, and 9 (19%) had no intervention. Among those who underwent surgical resection, 25 (66%) had maximum safe resection, 10 (26%) had total resection, 2 (5%) had subtotal resection, and 1 (3%) had only biopsy. Within 12 months, 10 (21%) of the CYPs expired. Further details are available in a previous study by our research group [21].

### Sociodemographic parental factors and financial burden of the disease

Eighteen (72%) mothers and 21 (84%) fathers were aged older than 35 years. The educational status of 11 (44%) mothers and 18 (72%) fathers was higher than or equal to secondary. Twenty one (84%) of the parents were married, while the remaining 4 (16%) were either widowed or divorced. In 19 (76%) of the households, fathers were the sole bread earners. The median household monthly income was 141 (71–442) USD. (Table 1)

**Table 1:** Sociodemographic parental factors of children and young people (5–21 years) with primary brain tumor (*n* = 25)

Characteristics	*n* (%)
**Age of Mothers’ (in year)**	
25–34 ≥ 35 **Mean age of Mothers’ (SD)**	7 (28) 18 (72) 38.7 (6.5)
**Age of Fathers (in years)**	
25–34 ≥ 35 **Mean age of Fathers (SD)**	4 (16) 21 (84) 42.8 (8.3)
**Marital status**	
Married Others (Widower, Widow, Divorce)	21 (84) 4 (16)
**Educational status of Mothers’**	
No formal education Primary Secondary Higher Secondary and above **Median Years of Mothers’ education (IQR)**	6 (24) 3 (12) 5 (20) 11 (44) 10 (0.5–14)
**Education status of Fathers**	
No formal education Primary Secondary Higher Secondary and above **Median Years of Fathers education (IQR)**	4 (16) 0 (0) 3 (12) 18 (72) 12 (10–15)
**Working status of the Parents**	
Only Father Working Only Mothers’ Working Both Father and Mothers’ Working Both Father and Mothers’ Not Working	19 (76) 2 (8) 1 (4) 3 (12)
**Household Monthly Income (in USD/PKR)**	
≤ 53/14,999 53–159/14,999-44997 159–318/44,997–89,994 318–3600/89994-1018800 **Median Household Monthly Income (IQR)**	5 (20) 9 (36) 4 (16) 7 (28) 141 (71–442)/39,903 (20,093-125,086)

Current conversion rate of USD is 283 PKR

When parents were asked about the financial impact of their CYP illness, only 1 out of 25 reported an unexpected hospitalization, and similarly, only 1 out of 25 reported quitting their job. Approximately 11 (44%) of the patients covered the CYP treatment expenses out of pocket, while 14 (56%) received financial assistance through AKUH welfare, Zakat, or insurance.

### Mean difference in quality of life and family functioning scores of mothers (pre-treatment versus post-treatment)

An increase in daily activity scores was found at 12 months posttreatment (mean difference = 10.3; 95% CI = 0.3, 20.3). However, there was no statistically significant difference in the remaining QoL domains (Table 2).

**Table 2:** Mean difference in quality of life and family functioning scores of mothers’ of children and young people (5–21 years old) with primary brain tumors 12 months post-treatment (*n* = 25)

Quality of Life domains	Mean Pre-treatment Scores (SD)	Mean Post-treatment Scores (SD)	Mean difference of QoL Scores (95% CI)
**Parent Functioning**			
Total Score	81.6 (13.3)	79.4 (17.3)	− 2.2 (-8.6,4.2)
Parent HRQOL Summary Score	80.0 (13.9)	78.9 (17.5)	− 1.1 (-7.8,5.7)
Physical Functioning	79.8 (13.4)	81.7 (16.5)	1.8 (− 5.2,8.9)
Emotional Functioning	74.2 (19.1)	71.8 (23.0)	- 2.4 (-11.9,7.2)
Social Functioning	86.0 (13.9)	81.5 (20.5)	- 4.5 (-14.6,5.6)
Cognitive Functioning	81.2 (21.6)	80.8 (25.9)	- 0.4 (-11.6,10.8)
Communication	94.3 (17.3)	96.3 (11.3)	2.0 (-3.2,7.2)
Worry	72.6 (23.8)	56.6 (34.4)	-16.0 (-33.4,1.4)
**Family Functioning**			
Family Summary Score	86.5 (17.5)	88.4 (22.4)	1.9 (-5.3,9.1)
Daily Activities*	74.7 (22.6)	85.0 (25.6)	10.3 (0.3, 20.3)
Family Relationships	93.6 (18.1)	90.5 (25.1)	- 3.1 (-11.6, 5.4)

*Significant at *p* value < 0.05 based on paired t test

### Regression analysis

On univariate analysis, there was a statistically significant decline in mothers’ mean 12-month QoL and family functioning scores in those whose CYPs had posttreatment seizures, had hydrocephalous and shunt placement, and had a high financial burden associated with CYP illness. However, there was a statistically significant improvement in the mean 12-month QoL and family functioning scores among those whose CYPs had high QoL scores, high verbal intelligence scores, high processing speed scores and high perceptual reasoning scores. However, there was no significant association of maternal QoL and family functioning with parental factors i.e., educational status, household monthly income and child’s factors i.e., age of the child, type of treatment, grade of tumor, tumor location and tumor size.

On multivariable analysis, a significant decrease in mothers’ mean 12-month QoL and family functioning scores was associated with CYP having posttreatment seizures (beta= -10.2; 95% CI: -18.4 to -2.0), and financial burden associated with the CYP’s illness (beta= -0.3; 95% CI: -0.4 to -0.1). However, in those patients in whom CYPs had higher posttreatment quality of life scores (beta = 0.4; 95% CI = 0.1, 0.6) and higher posttreatment verbal intelligence scores (beta = 0.1; 95% CI = 0.01, 0.3), the mothers’ QoL and family functioning scores were significantly greater (Table 3**).** No significant confounders were found.

**Table 3:** Factors associated with change in mean total quality of life scores for mothers’ of children and young people (5–21 years) with primary brain tumors (*n* = 25)

Characteristics	Unadjusted β coefficient (95% CI)	Adjusted β coefficient (95% CI)
**Surgery intervention**		
No intervention Biopsy Total Resection Subtotal resection Maximum safe Resection	(Reference) -17.6 (-43.9,8.7) 9.2 (-0.7,19.2) -15.7 (-32.1,0.8) 3.6 (-4.9,12.1)	-
**Post-treatment Seizures**		
Yes No	-12.4 (-25.6,0.7) (Reference)	**-10.1 (-18.4, -2.0**)
**Hydrocephalous**		
Yes No	-7.0 (-14.2,0.2) (Reference)	-
**Shunt Placement**		
Yes No	-6.8 (-14.0,0.5) (Reference)	-
**Household Monthly Income (in USD/PKR)**		
≤ 53/14,999 53–159/14,999-44997 159–318/44,997–89,994 318–3600/89994-1018800	-13.4 (-24.5, -2.3) - 9.8 (-18.7, -0.8) - 1.8 (-13.5,9.7) (Reference)	-
**Pre-treatment financial burden of the CYP’s disease**	-0.2 (-0.3, -0.1)	-
**Post-treatment financial burden of the CYP’s disease**	-6.5 (-13.8, 0.8)	**-0.2 (-0.4, -0.1)**
**Pre-treatment Qol scores of CYP**	0.4 (0.1, 0.8)	-
**Post-treatment Qol scores of CYP**	0.7 (0.4, 1.0)	**0.4 (0.1, 0.6)**
**Pre-treatment verbal intelligence scores of CYP**	0.2 (-0.01, 0.3)	-
**Post-treatment verbal intelligence scores of CYP**	0.3 (0.2, 0.5)	**0.1 (0.01, 0.3)**
**Pre-treatment processing speed scores of CYP**	0.2 (-0.1, 0.5)	-
**Post-treatment processing speed scores of CYP**	0.3 (-0.02, 0.6)	-
**Post-treatment perceptual reasoning scores of CYP**	0.3 (0.1, 0.5)	-

CI: confidence interval

QoL: Quality of life

USD: United states dollar

PKR: Pakistani rupee

## Discussion

Our prospective cohort study aimed to determine the factors associated with the mean change in QoL and family functioning of the primary caregivers of CYP patients with PBTs at 12 months posttreatment.

The findings of our study showed that the QoL and family functioning of mothers were predicted by the cognition status of CYPs. These findings were consistent with those of Hocking et al., who indicated that survivors’ neurocognitive functioning across all cognitive domains was related to mothers’ reported family functioning [8]. Peterson and Drotar’s model of childhood cancer survivorship depicts a significant correlation between the neurocognitive functioning of cancer survivors and family functioning support [40]. The intensity of neurocognitive deficits in CYP plays a crucial role in caregivers’ lives, placing increased demands on them and potentially straining parental and family resources. This strain may impede effective support for the neurocognitive and developmental needs of the child [8]. Strengthening initiatives focused on improving the cognitive functioning of CYP could play a pivotal role in enhancing the QoL of their caregivers and alleviating the overall burden on their families [41, 42]. 

Our study also revealed a significant association between the QoL of mothers and the QoL of their CYPs. Our findings were consistent with the results of Buchbinder et al., who highlighted a link between adverse physical and mental health outcomes in parents and the mental health of their children [7]. Prolonged treatment, recovery, and rehabilitation pose challenges to patients’ reintegration into normal life, contributing to an overall low QoL [43, 44]. Moreover, PBT survivors have a greater incidence of physical limitations than do those with other types of cancer [45]. Fuemmeler et al. and Chien et al. further support our observations, indicating that caregivers of children with PBT experience elevated levels of distress and lower QoL in both the physical and psychological domains [46, 47]. Assessment and treatment of caregiver needs are now standard of care in pediatric oncology [48], and tailoring support services to caregivers can enhance the overall functioning of the family, in turn empowering a PBT survivor to attain her/his maximum developmental potential [49]. 

We found a significant decrease in the QoL and family functioning scores of mothers whose CYPs had posttreatment seizures. Our findings are consistent with those of Thurman et al., who identified seizures among children as potential contributors to parental anxiety [50]. Cognition and behavioral changes in individuals with epilepsy have a multifactorial etiology, including epilepsy itself, treatment of epilepsy (with antiepileptic drugs or surgery), response to epilepsy (such as stigma, social marginalization, and familial dynamics), and any concurrent brain dysfunction and/or damage [51, 52], causing a substantial impact on the health-related quality of life (HRQoL) of parents [53, 54]. Furthermore, Fastenau et al. reported that seizures may result in academic difficulties [55], and attendance problems at school [56]. A systematic review meta-synthesis by Zhichao et al. [57] included 13 studies that explored the experiences and needs of caregivers of children with seizures. Over 50% of family members caring for children with seizures experience stigma, which negatively impacts caregivers’ mental health, as indicated by shame, low self-esteem, anger, and disorder disclosure [58]. The majority of caregivers reported a high burden of their child’s disease, socially, emotionally, functionally, and economically [59]. Yu Z et al. showed that the prevalence of anxiety and depression in caregivers of children who experienced seizures was 25% and 23.5%, respectively, suggesting that healthcare providers should pay attention to the psychological and emotional symptoms of caregivers [57]. Healthcare workers need to develop interventions to reduce the burden of caregivers, improve their mental health status, provide them with disease-related information and enhance their caring capacity [57].

The QoL and family functioning of mothers significantly declined at the 12-month reassessment due to the high costs associated with CYP treatment in our study. A similar finding was reported by Innasimuthu et al. from India [60]. Comparing stress levels across health systems with diverse financial models poses inherent challenges. Andersen et al. from the USA drew similar conclusions to ours, highlighting that different treatment modalities, including surgery, chemotherapy, or radiation therapy, not only increase the risk of side effects but also impose a significant financial burden on parents of children with PBTs [12]. The financial burden on parents plays a crucial role, with a significant proportion bearing the treatment costs out of their own pockets [61]. Similarly, in our study, 48% of the parents had to cover the expenses for CYP treatment out of pocket. Financial constraints pose a significant burden for many families in Pakistan. The average monthly income of middle-class households is approximately $230 [62]. The mean monthly cost per patient for cancer care at a private tertiary care hospital exceeds this amount by more than five times. The majority of these expenses are shouldered by the patient’s family [63]. In a study from Pakistan on adult brain tumors, the patients reported financial constraints attributable to treatment costs. Few mentioned becoming “hand-to-mouth” since the start of treatment, and well-to-do patients expressed their concern about exorbitant expenses [20]. To address financial burdens, the implementation of proactive measures such as patient assistance programmes at the institutional level can be effective. These programs, which attract support from multiple sponsors, offer tailored assistance based on each patient’s specific financial needs [64]. Within AKU, there is a charitable society responsible for collecting and disbursing zakat. It provides assistance to the most underprivileged members of society without any discrimination or compromise on the quality of care [65]. Moreover, government-led initiatives, such as Punjab’s health card system in Pakistan, provides a swift and dignified means to reduce out-of-pocket expenses for underprivileged people, enhancing healthcare access [20]. Similar programs should be initiated across the country.

### Limitations

This study has several limitations. First, the relatively small sample size due to loss to follow-up. However, the study retained adequate statistical power even though loss to follow-up was observed in 23/48 CYPs and their primary caregivers because sample size was inflated by 65% to account for potential loss to follow-up. Therefore, none of the characteristics were underpowered.

Second, the primary caregivers QoL and family functioning were assessed only before treatment and at 12 months after treatment. However, certain cognitive deficits of CYP may not have been apparent during the 12-month follow-up, and could potentially emerge beyond 12 months after the start of treatment [66]. Thus, it is plausible that a more extended follow-up duration might have unveiled significant shifts in neurocognitive outcomes.

Finally, the use of nonprobability purposive sampling technique introduces selection bias and limits the generalizability. In our study, patients who did not seek medical attention or treatment at the hospital might have been excluded from the study. Furthermore, individuals with more severe symptoms or advanced stages of brain tumors are more likely to seek treatment at the hospital, potentially leading to an overrepresentation of such cases in the sample. Moreover, by exclusively recruiting participants from two hospitals, our sample might not adequately represent those seeking care at other healthcare settings.

### Conclusions and future recommendations

We found a significant decrease in maternal QoL in mothers who had a high financial burden and whose CYP had posttreatment seizures. However, those whose CYPs had higher posttreatment verbal intelligence scores and quality of life scores had significantly greater QoL scores. Identification of the factors that influence parental QoL could enable the development of targeted strategies to alleviate stressors and improve the overall quality of life for mothers and their children who are at high risk. However, the limited size of our sample restricts the generalizability of our study findings. The next step would be ensuring internal consistency with a new cohort of CYP patients with PBTs and testing it in studies outside the country.

### Study implications

The identification of factors influencing the quality of life (QoL) of primary caregivers in the context of primary brain tumors in children and young people has significant implications for healthcare and support services. By determining these factors, the study facilitates the development of tailored support services, such as enhanced mental health support, interventions addressing financial stress, and coping strategies. This research contributes to education and awareness programs for healthcare providers and the public, emphasizing the challenges faced by primary caregivers. Future research priorities can be shaped to guide a focus on important factors in primary caregivers’ QoL outcomes. The emphasis on family-centered care, consideration of long-term implications, and the development of social support initiatives can collectively contribute to more comprehensive and empathetic care for families navigating life.

### Electronic supplementary material

Below is the link to the electronic supplementary material.

Supplementary Material 1

## Data Availability

The data that support the findings of this study are available from the corresponding author upon reasonable request.

## Declarations

### ClinicalTrials.gov identifier

NCT05709522.

## Abbreviations

AKUH: Aga khan university hospital
CI: Confidence Interval
CV: Coefficient of variation
CVI: Content validation index
CYP: Children and young people
IQR: Inter quartile range
JPMC: Jinnah postgraduate medical center
LMICs: Low-and middle-income countries
MRI: Magnetic resonance imaging
PBTs: Primary brain tumor
PC: Proportionate change in means
PedsQL: Pediatric quality of life inventory
QoL: Quality of Life
RA: Research Assistant
RPM: Raven’s progressive matrices
SITS R3: Slosson Intelligence tool, Revised 3rd edition
SD: Standard deviation
SES: Socioeconomic Status
USD: United states dollar
VAS: Visual analog scale
WISC-V: Wechsler Intelligence Scale for Children
WAIS-IV: Wechsler Adult Intelligence Scale
