# Supplementary material for: Factors associated with changes in the quality of life and family functioning scores of primary caregivers of children and young people with primary brain tumors in Karachi, Pakistan: a prospective cohort study
Source: BMC Pediatr. 2024 Jun 8;24:389. doi: 10.1186/s12887-024-04867-z (PMC11161978; doi:10.1186/s12887-024-04867-z)
Supplement: Supplementary file 1 — Supplementary Material 1 [file 12887_2024_4867_MOESM1_ESM.docx]

| **SUPPLEMENTAL TABLE 1 Internal consistency of PedsQL Family Impact Module (n=48)** | | | | | | | |
| --- | --- | --- | --- | --- | --- | --- | --- |
| PedsQL Family Impact Module | Number of Items | Cronbach’s alpha | Median | Mean | SD | Floor (%) ^a^ | Ceiling (%) ^b^ |
| **Parent Functioning** | | | | | | | |
| Total Score | 36 | 0.9* | 82.3 | 79.7 | 12.8 | 2.1 | 2.1 |
| Parent HRQOL  Summary Score | 20 | 0.9 * | 78.8 | 76.7 | 14.3 | 2.1 | 2.1 |
| Physical Functioning | 6 | 0.7* | 77.1 | 77 | 12.9 | 2.1 | 6.3 |
| Emotional Functioning | 5 | 0.79 * | 72.5 | 69.9 | 18.6 | 6.3 | 2.1 |
| Social Functioning | 4 | 0.5 * | 87.5 | 83.1 | 15.4 | 4.2 | 25 |
| Cognitive Functioning | 5 | 0.9 * | 90 | 77.9 | 22.9 | 2.1 | 33.3 |
| Communication | 3 | 0.8 * | 100 | 93.8 | 14.5 | 2.1 | 77.1 |
| Worry | 5 | 0.8* | 70 | 71.4 | 22.5 | 2.1 | 22.9 |
| **Family Functioning** | | | | | | | |
| Family Summary  Score | 8 | 0.9 * | 90.6 | 87.1 | 15.2 | 2.1 | 29.2 |
| Daily Activities | 3 | 0.9 * | 75 | 75.2 | 22.1 | 2.1 | 29.2 |
| Family Relationships | 5 | 0.9 * | 100 | 94.3 | 14.3 | 2.1 | 79.2 |
| *SD Standard deviation*  **Significant at p-value<0.01by reliability analysis*  *Percentage of patients with highest recorded score a*  *Percentage of patients with lowest recorded score b* | | | | | | | |
